# Runs of Homozygosity Detection and Selection Signature Analysis for Local Goat Breeds in Yunnan, China

**DOI:** 10.3390/genes15030313

**Published:** 2024-02-28

**Authors:** Chang Huang, Qian Zhao, Qian Chen, Yinxiao Su, Yuehui Ma, Shaohui Ye, Qianjun Zhao

**Affiliations:** 1 College of Animal Science and Technology, Yunnan Agricultural University, Kunming 650201, China; brookhuang2000@163.com (C.H.); zhaoqian12242021@163.com (Q.Z.); 2 State Key Laboratory of Animal Biotech Breeding, Institute of Animal Sciences, Chinese Academy of Agricultural Sciences (CAAS), Beijing 100193, China; chenqian602141@163.com (Q.C.); suyingxiao_00@163.com (Y.S.); mayuehui@caas.cn (Y.M.)

**Keywords:** runs of homozygosity, heterozygosity, local goat breeds, candidate genes, Yunnan province

## Abstract

Runs of Homozygosity (ROH) are continuous homozygous DNA segments in diploid genomes, which have been used to estimate the genetic diversity, inbreeding levels, and genes associated with specific traits in livestock. In this study, we analyzed the resequencing data from 10 local goat breeds in Yunnan province of China and five additional goat populations obtained from a public database. The ROH analysis revealed 21,029 ROH segments across the 15 populations, with an average length of 1.27 Mb, a pattern of ROH, and the assessment of the inbreeding coefficient indicating genetic diversity and varying levels of inbreeding. iHS (integrated haplotype score) was used to analyze high-frequency Single-Nucleotide Polymorphisms (SNPs) in ROH regions, specific genes related to economic traits such as coat color and weight variation. These candidate genes include *OCA2* (OCA2 melanosomal transmembrane protein) and *MLPH* (melanophilin) associated with coat color, *EPHA6* (EPH receptor A6) involved in litter size, *CDKAL1* (CDK5 regulatory subunit associated protein 1 like 1) and *POMC* (proopiomelanocortin) linked to weight variation and some putative genes associated with high-altitude adaptability and immune. This study uncovers genetic diversity and inbreeding levels within local goat breeds in Yunnan province, China. The identification of specific genes associated with economic traits and adaptability provides actionable insights for utilization and conservation efforts.

## 1. Introduction

Believed to have been domesticated around 10,000 years ago, the goat (*Capra hircus*) species ranks among the earliest livestock to be tamed [1,2]. Since the Neolithic era, goats have been pivotal in agriculture, providing fiber, milk, and meat [3]. The evaluation of genetic diversity for native goat breeds is not only essential for the conservation and utilization of animal resources but also crucial for understanding the basis of key traits such as disease resistance, productivity, and adaptability [4]. It also sheds light on their evolutionary history and domestication process and ensures the sustainability and adaptability of goat populations to changing environments [5].

Yunnan province is located in southwestern China, bordering the countries of Myanmar, Laos, and Vietnam. It is characterized by its complex topography, diverse climate, and abundant biodiversity and is the home to the highest concentration of ethnic minorities [6,7]. During long-term natural and artificial selection, various local goat breeds in Yunnan province emerge with divergent traits. In this study, we focused on ten goat breeds from Yunnan, all listed in China’s National Livestock and Poultry Genetic Resources Catalog. For example, the Maguan polled goat breed is a rare and valuable genetic resource with the polled trait since its population size has decreased dramatically in recent years. Longling yellow goat is a unique breed with high meat quality and disease resistance and is included in the Yunnan Province Livestock and Poultry Genetic Resources Protection List to better safeguard this exceptional breed. This situation highlights the need for comprehensive genetic studies, such as those employing microsatellite markers to assess genetic diversity in Yunnan indigenous goat populations [7], underscoring the importance of conservation and utilization for these unique goat breeds in Yunnan. However, a comprehensive analysis of the genome-wide patterns of homozygosity and selection signatures in these breeds is still lacking.

In diploid genomes, ROH are continuous homozygous segments of the DNA sequence [8,9]. Demographic events like population bottlenecks, genetic drift, and inbreeding primarily contribute to the formation of ROH [10,11]. These homogenous DNA segments are instrumental in the analysis of inbreeding in livestock species [12,13,14]. In livestock, the length of ROH segments serves as an indicator of the level of inbreeding, with longer ROH segments denoting recent inbreeding events and shorter segments indicating inbreeding that occurred earlier in the generations [15,16]. ROH islands, first identified by Nothnagel et al. [17], provide insights into exploring genomic regions with significant inbreeding in populations [18]. The ROH islands were observed in various livestock species, including cattle [19], pigs [20], horses [21], sheep [22], and goats [23]. However, there has been limited research on the identification of ROH in local goat breeds from Yunnan province, China.

Recent studies have played a crucial role in assessing genetic diversity and inbreeding levels in goat populations utilizing SNP microarrays and whole-genome resequencing methods. These studies emphasize the differences in genetic diversity across various breeds and point out the considerable inbreeding present in some breeds [13,24,25]. Compared to microarray methods, the whole-genome resequencing method offers a more comprehensive detection of genetic variations, providing deeper insight into the genetic landscape [26,27,28]. Furthermore, the iHS analysis applied to whole-genome resequencing and SNP microarray data have proven effective in identifying genomic regions under positive selection in goats; this approach facilitated the identification of genes associated with key traits such as productivity, disease resistance, and climate adaptability [4,29,30].

In this study, we focused on ten goat breeds from Yunnan, all listed in China’s National Livestock and Poultry Genetic Resources Catalog. We investigated the genome-wide patterns of ROH and estimated the genomic inbreeding coefficients in these populations. Additionally, our research also identified candidate genes associated with important traits through selection signal analysis of SNPs within ROH regions. This research reveals the genetic diversity, inbreeding level, and specific set of genes with significant selection signals in local goat breeds from Yunnan province, which provide useful information for the conservation and utilization of these goat breeds.

## 2. Materials and Methods

### 2.1. Ethics Statement

The experimental procedures involving animals received approval from both the Chinese Ministry of Agriculture and the Institute of Animal Science at the Chinese Academy of Agricultural Sciences. The ethical committee of the Institute granted approval for animal welfare under the reference number IASCAAS-AE-03. Approval was given on 1 September 2014.

### 2.2. Sample Collection, DNA Extraction, and Sequencing

Ear marginal tissue samples were collected from a total of 98 goats, including 94 individuals from Yunnan province (Fengqing Polled Black goat, 8; Guishan goat, 10; Longling Yellow goat, 10; Luoping Yellow goat, 10; Maguan Polled goat, 10; Mile Red Bone goat, 6; Ninglang Black Head goat, 10; Weixin White goat, 10, Yunling goat, 10; Zhaotong goat, 10) and 4 samples with Laoshan Dairy goat. The samples were stored in 75% alcohol and then stored at −80 °C. In addition, we downloaded 24 goat resequencing data from NCBI (National Center for Biotechnology Information (nih.gov)) (Supplementary Table S1). The feeding regimens for these goats were designed to fulfill their nutritional needs, tailored to their body weight and production performance, ensuring the collection of representative biological samples under optimal physiological conditions.

DNA extraction was conducted using a Wizard^®^ Genomic DNA Purification Kit (Promega, Madison, WI, USA), which involves preparing the sample with EDTA and Nuclei Lysis Solution, digesting the tissue with Proteinase K, purifying the DNA through precipitation and centrifugation, and finally, rehydrating the DNA pellet for storage. The concentration and purity of DNA samples were quantified using a NanoDrop 2000 spectrophotometer (Thermo Fisher Scientific Inc., Waltham, MA, USA). Only DNA samples with a concentration of more than 20 ng/µL and a purity ratio (A260/A280) of 1.8–2.0 were used for library construction and sequencing.

The sequencing libraries were constructed according to the manufacturer’s instructions (Illumina Lnc., San Diego, CA, USA) and sequenced on the DNBSEQ-T7 platform (Shenzhen MGI Co., Ltd., Shenzhen, China) with PE150 model. Briefly, the sequencing libraries were generated following the protocol of the NEBNext Ultra II DNA Library Prep Kit for Illumina (New England Biolabs, Ipswich, MA, USA). Initially, DNA was sonicated to a target size of 300 bp, followed by end repair, A-tailing, and adapter ligation with NEBNext adapters. Size selection was conducted using AMPure XP beads (Beckman Coulter, Brea, CA, USA), and the fragments were PCR amplified. Library quality and concentration were evaluated using the Qubit 2.0 Fluorometer (Thermo Fisher Scientific, Waltham, MA, USA) and Agilent 2100 Bioanalyzer (Agilent Technologies, Santa Clara, CA, USA).

### 2.3. Resequencing Data Processing, SNP Calling, and Annotation

Quality filtering and trimming of the raw sequencing reads were performed using NGS QC Toolkit (Version: 2.3.3) [31]. Then, the clean reads aligned with ARS1.2 (GCF_001704415.2) goat reference genome, using the BWA-MEM (Version: 0.7.12) [32,33]. The alignment output was formatted into BAM format and sorted by SAMtools (Version 1.3.1) [34]. Duplicate reads were removed using SAMtools. The genome-wide SNPs were identified with HaplotypeCaller and then merged by GenomicsDBImport and GenotypeGVCFs using GATK (Version 3.7) [35]. SNPs were annotated using SnpEff software (Version 4.0) [36] based on the goat reference genome ARS1.2.

### 2.4. ROH Analysis and Classification

ROHs were detected across autosomes for each samples using PLINK (Version 1.90b) [37] (--homozyg-density 30 --homozyg-gap 1000 --homozyg-kb 500 --homozyg-snp 50 --homozyg-window-het 1 --homozyg-window-snp 30 --homozyg-window-missing 5 --homozyg-window-threshold 0.05). ROHs were divided into 4 classes based on length: (A) 500 kb to 1 Mb, (B) 1 Mb to 2 Mb, (C) 2 Mb to 4 Mb, and (D) >4 Mb.

### 2.5. Genomic Inbreeding Coefficients

Genomic inbreeding coefficients were determined utilizing PLINK. The calculation method of *F_ROH_* was as follows: *F_ROH_* = (*L_ROH_*/*L_AUTO_*) [38], where *L_ROH_* represents the sum length of an individual’s ROH in the genome, and *L_AUTO_* is the specific length of the autosomal genome covered by SNPs of goat (2466.19 Mb) based on the goat reference genome ARS1.2.

*2.6. iHS Analysis and Gene Annotation*

The top 5% of SNPs with the highest occurrence frequency in each population were selected for further analysis [39,40]. The iHS for SNPs within high-frequency ROH regions was calculated for the local goat breeds from Yunnan province utilizing the rehh [41] R package (Version: 3.2.2). SNPs in the top 1% of iHS scores were designated as strongly selected sites [42]. SnpEff software (Version: 4.0) was used to annotate genes with strongly selected sites.

## 3. Results

### 3.1. Sequencing and Genetic Variation

Whole-genome resequencing was conducted on 94 goat samples from Yunnan province and four LSD samples (Table 1), generating a total data of 2.94 Tb, with an average depth of 10× per individual. The data of BEZ, ANG, BER, and BLB goat populations were downloaded from NCBI (Supplementary Table S1). A total of 11,603,450 SNPs were filtered for subsequent analysis in ROH.

### 3.2. Genomic Distribution of ROH and Inbreeding Coefficients

**Figure 1:**
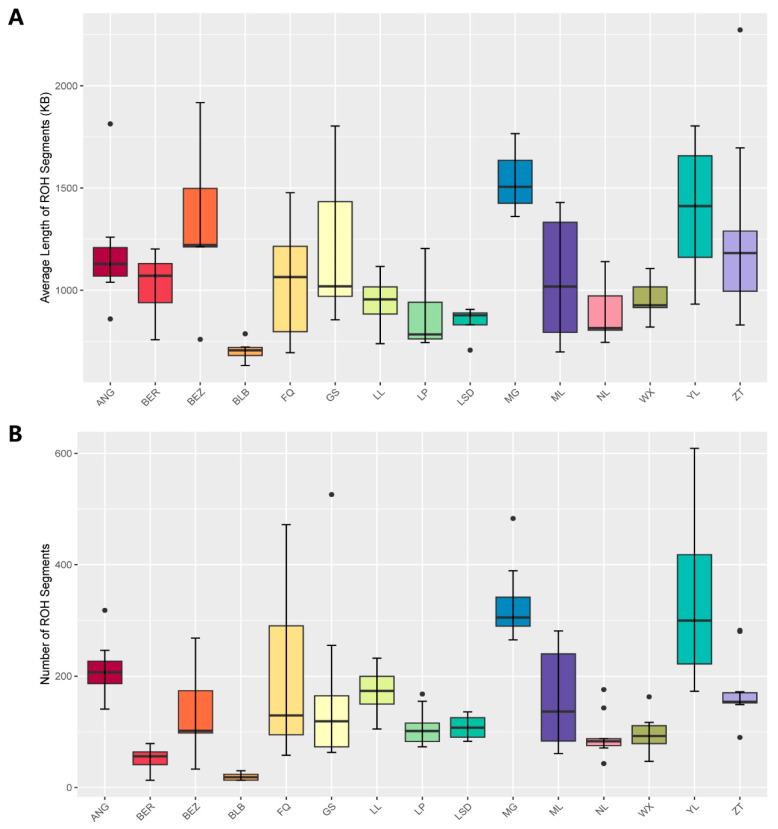
The distributions of ROH statistics per individual for 15 goat populations, including ANG (n = 8), BER (n = 5), BEZ (n = 5), BLB (n = 6), FQ (n = 8), GS (n = 10), LL (n = 10), LP (n = 10), LSD (n = 4), MG (n = 10), ML (n = 6), NL (n = 10), WX (n = 10), YL (n = 10), and ZT (n = 10). (**A**) The length of ROHs per individual. (**B**) The number of ROHs per individual.

Across 15 goat populations, we assessed ROHs on a genome-wide scale spanning 29 autosomes. The analysis revealed 21,029 ROHs, averaging 172 per individual. These segments ranged from 0.5 to 21 Mb in length, with an average segment size of 1.27 Mb across all autosomes (Table 1, Supplementary Tables S2 and S3). Notably, the YL and MG goat breeds exhibited the longest average ROH segment length at 492 Mb and 507 Mb, double the length of the breed of ANG (257 Mb), which underwent intensive selection in wool production (Figure 1). The breeds of local goat FQ (246 Mb), ZT (243 Mb), GS (222 Mb), and ML (193 Mb) from Yunnan were similar to the length of ANG (Figure 1). In contrast, the BLB goats showed the lowest homozygosity, with 117 ROH events with an average length of 14 Mb (Figure 1). Furthermore, MG goats had the most extensive genome coverage by ROH (corresponding to ~20.55% of goat autosomes genome) (Figure 1). Our study revealed breed-specific variations in the frequency and dimensions of ROH events, with the MG breed showing a greater average ROH length compared to other breeds.

**Figure 2:**
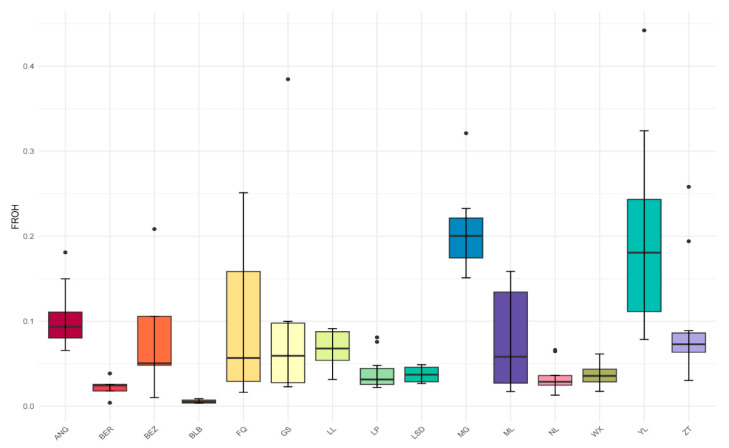
Values of *F_ROH_* for each population.

To obtain the inbreeding coefficients of each population, the *F_ROH_* was used to estimate the value of inbreeding coefficients. The *F_ROH_* values for each population ranged from 0.02 to 0.21 (Figure 2). In the native goat breeds from Yunnan province, the breed MG exhibited the highest average *F_ROH_* at 0.21, closely followed by the YL goat breed at 0.20 (Figure 2). Conversely, the breed of NL displayed the lowest average *F_ROH_* at 0.03 (Figure 2). Notably, the inbreeding coefficients of local goat breeds from Yunnan were generally higher compared to those in the BER, BLB, and LSD breeds. BLB (0.05), with the fewest number of ROH events and the shortest ROH segment length, showed the lowest inbreeding coefficient. Interestingly, despite having a higher count of ROH events, the breed of ZT goat exhibited relatively low levels of *F_ROH_*.

### 3.3. Genomic Patterns of Homozygosity

**Figure 3:**
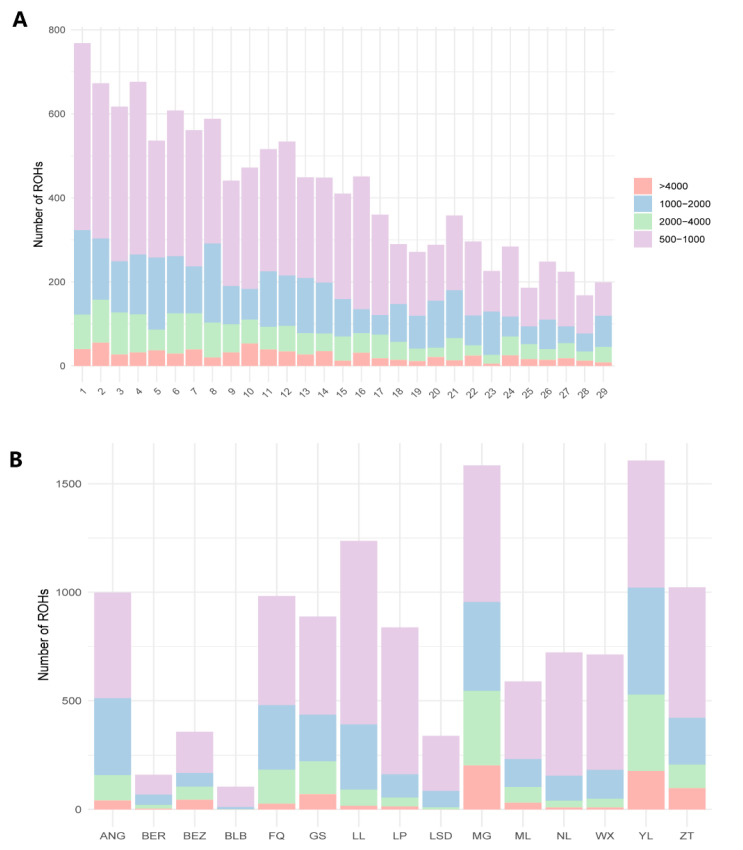
Total number of ROH in 15 goat populations. (**A**) Number of ROH in chromosome with different size classes. (**B**) The number of ROH belonging to four size classes, including 500 kb to 1 Mb, 1 Mb to 2 Mb, 2 Mb to 4 Mb, and >4 Mb for each of the different populations.

To investigate the patterns of ROH in each goat population, we categorized ROH segments into four size classes: (A) 500 kb to 1 Mb, (B) 1 Mb to 2 Mb, (C) 2 Mb to 4 Mb, and (D) >4 Mb. Notably, a significant number of ROHs were observed on chromosome 1, while chromosome 28 exhibited the fewest (Figure 3A). We provide a detailed distribution of ROHs across each chromosome, including the count and classification of ROHs in different size ranges (Figure 3A).

The total number and each category of ROHs for populations are depicted in Figure 3B. Particularly, breeds MG and YL displayed the highest total number of ROH segments (greater than 4 Mb), contrasting with the BLB breed, which showed the lowest number in this category. The analysis also indicated that ROH segments in categories A and B, predominantly featuring segments ranging from 500 kb to 1 Mb, were predominant across all populations (Figure 3B). Additionally, our study found that ROH segments between 2 and 4 Mb are relatively abundant in the genomic landscape of local goat breeds from Yunnan province, particularly in the YL and MG breeds. These results reveal breed-specific differences in ROH distribution, providing insights into the genetic diversity of these breeds.

### 3.4. iHS Selection Signature Analysis

To investigate the effect of selection in local goat breeds from Yunnan province, we explored the distribution of ROH throughout the genome. The frequency of SNPs within ROH regions in each breed was quantified, the top 5% SNPs with high frequency in ten local goat breeds from Yunnan province were annoted, with the software SnpEff (Version: 4.0). To further refine the selection of candidate genes, a haplotype based on the iHS method was then used to detect selection signals in the top 5% of SNPs with high frequency across 10 native goat breeds from Yunnan, and these were then annotated with the software of SnpEff.

**Figure 4:**
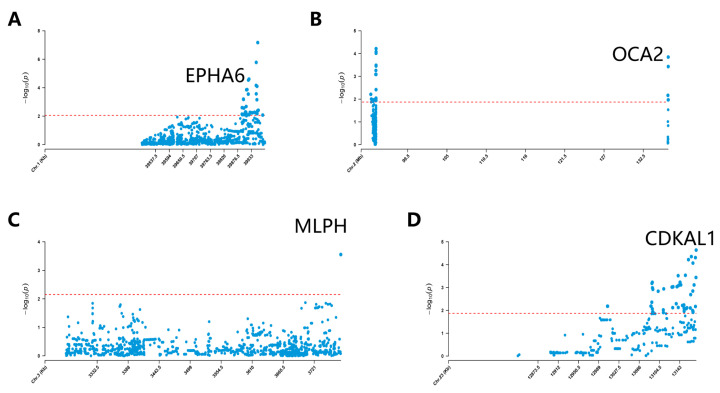
The loci with strong selection signals, as identified by the iHS, were annotated to specific genes. (**A**) *EPHA6* gene in GS breed, (**B**) *OCA2* gene in ML breed, (**C**) *MLPH* gene in GS breed, (**D**) *CDKAL1* gene in FQ breed. The dashed line represents the top 5% SNPs with high frequency in the local goat breed.

In all breeds analyzed in Yunnan province, we identified the top 1% of iHS values, which represented strong signals of selection; a total of 443 genes were annotated (Supplementary Table S4, Supplementary Figure S1). For instance, in the breeds of GS and ML, the *EPHA6* gene was annotated in the top 1% of iHS values (Figure 4A). This gene was known for its association with litter size, the rs402032081 variant of *EPHA6,* which was reported in Polish Mountain sheep [43]. Multiple strong selection signals on chromosome 2 in the breeds of LP, ML, and YL were annotated to the *OCA2* gene (Figure 4B), known for its association with skin pigmentation [44]. On chromosome 3 of both ML and GS goats, we identified the *MLPH* gene (Figure 4C), which was characterized as a candidate gene for dilute coat color in some goat breeds [45]. Additionally, *CDKAL1* (FQ) [46] and *POMC* (FQ) [47] were identified as related to weight variation (Figure 4D), LIM domain binding 1 (*LDB1*) and fibroblast growth factor 2 (*FGF2*) (NL) [48,49] were identified related to high-altitude adaptation (Table 2). Our research also revealed that a specific subset of genes shows pronounced selection signals throughout the local goat breeds from Yunnan province, highlighting the presence of breed-specific selective loci. This pattern suggests that each breed from Yunnan possesses a distinctive genetic identity, shaped by varying degrees of artificial and natural selection.

## 4. Discussion

The increase in homozygosity observed in specific genomic regions of livestock can be attributed to various factors, including population bottlenecks, genetic drift, and inbreeding, which collectively contribute to the heightened frequency of ROH and affect the genomic diversity within these populations and breeds. Many studies investigated the patterns of ROH and their correlation with inbreeding depression, particularly in relation to important traits in various goat breeds [53,54]. However, the patterns and distribution of ROH in local goat breeds from Yunnan province remain largely unexplored. In this study, whole-genome resequencing was utilized to explore the ROH patterns and distribution in native goat breeds from Yunnan.

The distribution of the number and length of ROH can reflect the genetic diversity within the studied populations. We observed variations in the total number and length of ROH among local goat breeds from Yunnan province (Figure 1), consistent with previous studies on cattle [55], sheep [56], and horses [57]. The local goat breeds from Yunnan, including MG, YL, and ZT, exhibited a higher number and length of ROH compared to the populations of BEZ, BLB, BER, and LSD (Figure 1), the variations of which possibly attributed to factors such as historical breeding practices, geographical isolation, or specific genetic characteristics unique to each breed, reflecting adaptation to local environmental conditions. The MG breed is endangered, exhibiting genetic degradation, a small effective population size, and the polled unique genetic characteristic factors that likely contribute to the accumulation of ROH segments (Figure 1). For YL goats, despite being distributed throughout Yunnan province, they suffered from a lack of systematic selective breeding and population protection. Consequently, these goats are characterized by their small size, slow growth and development, and low lamb survival rates, leading to increasingly evident population degeneration. Additionally, they exhibit a high number of long ROH segments (Figure 1), indicating elevated inbreeding coefficients (Figure 2).

A breed exhibited a higher inbreeding coefficient, indicating a potential reduction in genetic diversity and an increased frequency of deleterious genotypes [58]. Among these local goat breeds, MG and YL have higher *F_ROH_* than other breeds, which highlights the urgent need for targeted efforts to preserve MG genetic diversity and reverse the trend of population decline and genetic conservation and management strategies to mitigate the adverse effects of inbreeding and promote the sustainable development of the YL goat population. This is like the goat breeds of Mallorquina and Blanca de Rasquera, in which population size declines led to an increase in the frequencies of large-size ROH segments and the extent of inbreeding [59]. For the breeds of LP and ZT, a large number and length of ROH segments were observed, but their *F_ROH_* levels were relatively low. This pattern might result from the presence of a few individuals with higher inbreeding coefficients within the breeds.

Diverse patterns of ROH across different breeds offer insights into their genetic diversity and provide evidence for conservation. In our study, we observed that the longer autosomes contained a higher number of ROH. This finding aligns with previous research conducted on goats [13], sheep [60], and cattle [61]. These events can have profound effects on the genetic diversity and breed characteristics of the studied goat populations. For instance, inbreeding can lead to the accumulation of deleterious alleles, which can negatively impact the health and fitness of the population [62]. Conversely, it can also lead to the fixation of beneficial alleles, which can enhance certain breed characteristics. Notably, we categorized ROH segments into four classes according to their length and discovered that short ROH segments predominantly constitute the ROH in different breeds. As the length of ROH segments increases, their frequencies decrease, indicating that the ROH patterns can reflect the occurrence of inbreeding events in either a relatively recent or more accent generational context. This finding aligns with the results of ROH analysis in cattle populations from southern China [61]. Furthermore, our research revealed that in local Yunnan goat breeds, particularly in MG and YL breeds, the number of ROH segments exceeding 1 Mb was substantially higher than those ranging from 0.5 to 1 Mb. This pattern suggests that these breeds may have undergone a reduction in effective population size or experienced inbreeding, which needs further conservation measures for these breeds.

In breeding programs, particularly for local breeds facing challenges like low slaughter rates and limited market demand, managing genetic diversity and controlling the inbreeding coefficient is crucial for preserving unique traits and ensuring long-term sustainability [63]. The calculation of inbreeding coefficients using *F_ROH_* was identified as a highly accurate method for assessing inbreeding levels within a population [64]. For instance, the inbreeding level of American Angus cattle was accessed using ROH, homozygous-by-descent (HBD) segments, alongside traditional pedigree measures [65], which indicated the *F_ROH_* method was shown with higher accuracy for genetic diversity and inbreeding quantification. A study on a Large White pig population utilized *F_ROH_*, among other inbreeding coefficient estimations, reinforcing the significance of genomic approaches in understanding the genetic diversity of livestock populations [66]. This approach helps conserve and utilize local breeds, preserving their unique genetic traits and enhancing their contributions to biodiversity and agriculture. In light of our findings, we propose several conservation measures for local goat breeds, especially those with higher *F_ROH_* levels, which are at greater risk of inbreeding. Controlled breeding strategies, including the introduction of unrelated individuals to increase genetic diversity and regular genetic monitoring to track ROH and *F_ROH_* levels, are suggested. Establishing gene banks for preserving genetic material and ongoing research into their genetic characteristics are crucial for the breeds’ long-term survival and well-being.

Under diverse climates, geographical distributions, and artificial selection, the local goat breeds with distinctive traits from Yunnan province of southwest China have been shaped. In our study, the iHS method was utilized to detect selection signals in the top 1% of high-frequency SNPs across local goat populations. iHS is a widely used method for detecting positive selection in populations based on haplotype data. This analysis was focused on these top SNPs, aiming to identify potential selective signals. The NL breed from Yunnan province is situated in the middle section of the Hengduan Mountains. This region serves as a transitional area between the Qinghai–Tibet Plateau and the Yunnan–Guizhou Plateau, characterized by an average altitude of 2800 m. The iHS analysis revealed some genes were identified in the NL breed, which was reported to be associated with high-altitude adaptability, such as *LDB1* and *FGF2* [48,49]. It is suggested that the NL goats living at high altitudes may be experiencing natural selection pressure due to the environmental conditions of high altitudes. In local meat goat breeds, such as FQ and WX, iHS analysis results revealed the genes of *CDKAL1* and *POMC* linked to body weight [47,67], indicating potential selection for traits associated with meat production. Additionally, genes associated with coat color and skin pigmentation, such as *MLPH* (in GS and ML breeds) and *OCA2* (in LP, ML, and YL breeds), were identified [45,47]. Notably, the knockout of the *OCA2* gene in Astatotilapia calliptera was shown to lead to the absence of melanin [44]. The immune-related genes *CD53* (CD53 molecule) and *SSBP2* (single-stranded DNA binding protein 2) were identified in the GS goat breed, which plays a role in pathogen resistance and the regulation of mammary gland inflammation, thus indirectly influencing the efficiency and quality of milk production [68]. Moreover, the *MAGI2* (membrane-associated guanylate kinase, WW, and PDZ domain containing 2) gene, associated with reproductive traits in goats, was identified across six breeds (FQ, GS, LL, LP, NL, ZT). In dogs, *MAGI2* was reported to be associated with ovary formation during early embryonic development [69], which may play a significant role in goat reproductive traits.

In conclusion, our study provides valuable insights into the genetic diversity and structure of local goat breeds from Yunnan province, China. We identified variations in the number and length of ROH among these breeds, which can be attributed to factors such as historical breeding practices, geographical isolation, and breed-specific genetic characteristics. Our findings underscore the importance of implementing effective conservation strategies, particularly for breeds with higher *F_ROH_* levels, to preserve their genetic diversity and mitigate the effects of inbreeding. Furthermore, through iHS analysis, we identified candidate genes related to key traits, such as coat color, litter size, weight variation, high-altitude adaptability, and immunity. These findings contribute to the conservation and utilization of local goat breeds and enrich our broader understanding of livestock genetic diversity.

## Figures and Tables

**Table 1:** The descriptive statistics of ROH for 15 goat populations.

Code	Breed/Population	Sample Size	Total Number ^1^	Average Number per Individual	Total Length (Mb) ^2^	Average Length per Individual (Mb)
ANG	Angora goat	8	1709	214	2055	257
BER	Bermeya goat	5	253	51	272	54
BEZ	Bezoar	5	675	135	1043	209
BLB	Black Bengal goat	6	117	20	83	14
FQ	Fengqing Polled Black goat	8	1672	209	1964	246
GS	Guishan goat	10	1617	162	2217	222
LL	Longling Yellow goat	10	1737	174	1681	168
LP	Luoping Yellow goat	10	1069	107	985	99
LSD	Laoshan Dairy goat	4	434	109	369	92
MG	Maguan Polled goat	10	3288	329	5067	507
ML	Mile Red Bone goat	6	955	160	1155	193
NL	Ninglang Black Head goat	10	927	93	848	85
WX	Weixin White goat	10	953	95	906	91
YL	Yunling goat	10	3333	333	4918	492
ZT	Zhaotong goat	10	1750	175	2430	243

^1^ The total number of ROH events for each population. ^2^ Cumulative ROH event lengths per individual within each population.

**Table 2:** Genes identified by iHS selection signature analysis.

Gene Symbol	Gene Name/Description	Breed in This Study	Chromosome	Function	Reference
*OCA2*	OCA2 melanosomal transmembrane protein	LP, ML, YL	2	Coloration of the coat	[44]
*IGF2BP2*	insulin-like growth factor 2 mRNA binding protein 2	YL	1	Reproductive process and reproduction in goats	[50]
*CAMK2D*	calcium/calmodulin-dependent protein kinase II delta	GS	6	Protein serine/threonine kinase activity. embryo development	[51]
*CDKAL1*	CDK5 regulatory subunit associated protein 1 like 1	FQ	23	Chest depth measurements	[47]
*LDB1*	LIM domain binding 1	NL	26	High-altitude adaptation	[48]
*FGF2*	fibroblast growth factor 2	NL	17	High-altitude adaptation	[49]
*DGKB*	diacylglycerol kinase β	LL	14	Innate and adaptive immunity	[52]

## Data Availability

The data presented in this study are available upon request from the corresponding author.

## Supplementary Materials

The following supporting information can be downloaded at: https://www.mdpi.com/article/10.3390/genes15030313/s1, Supplementary Table S1: Information on Goat Samples Downloaded from the NCBI Database. Supplementary Table S2: Lists the detected ROHs. Each ROH is described by the chromosome, starting and ending SNP, the number of SNPs, and the length in kb. Supplementary Table S3: Summarizes the ROHs for each individual, including counts and total lengths. Supplementary Table S4: Genes annotated with the strongest selection signals in the top 1% overlapping among goat breeds from Yunnan province, China. Supplementary Figure S1: Genes identified by iHS selection signature analysis as overlapping among various goat breeds from Yunnan province, China.
