# Supplementary figures and images for: Runs of Homozygosity Detection and Selection Signature Analysis for Local Goat Breeds in Yunnan, China
Source: Genes (Basel). 2024 Feb 28;15(3):313. doi: 10.3390/genes15030313 (PMC10970279; doi:10.3390/genes15030313)

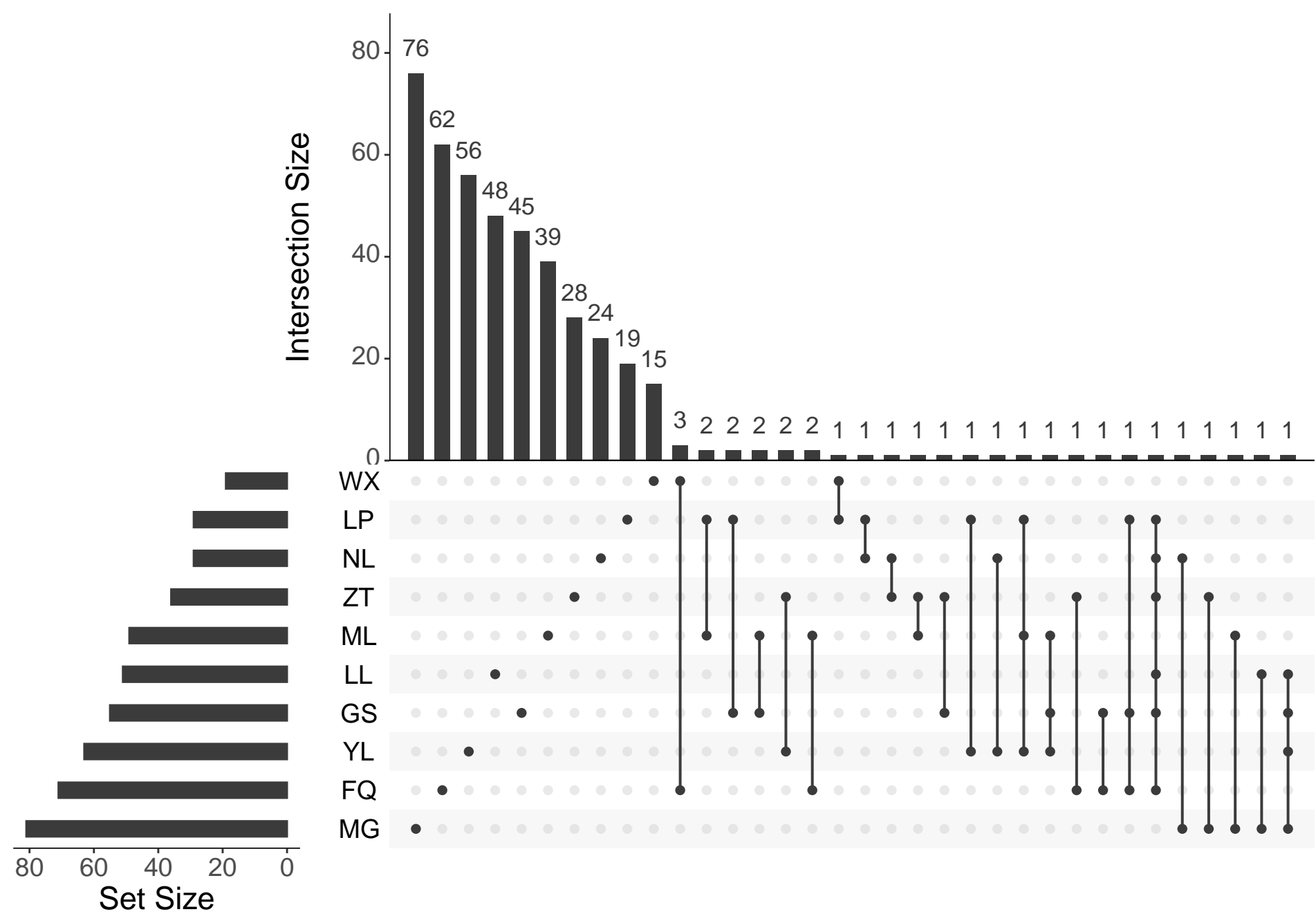

Supplement: Supplementary file 1 [file genes-15-00313-s001.zip › Supplementary Figure S1.pdf]
